# Tire shops in Miami-Dade County, Florida are important producers of vector mosquitoes

**DOI:** 10.1371/journal.pone.0217177

**Published:** 2019-05-20

**Authors:** André B. B. Wilke, Chalmers Vasquez, William Petrie, John C. Beier

**Affiliations:** 1 Department of Public Health Sciences, Miller School of Medicine, University of Miami, Miami, Florida, United States of America; 2 Miami-Dade County Mosquito Control Division, Miami, Florida, United States of America; University of Crete, GREECE

## Abstract

Human mobility in urban environments is a central part of urbanization and has determined the layout of how cities are projected, built and renovated. One of the most problematic issues of urbanization is how to properly dispose of used tires, considering the worldwide annual production of approximately 1.4 billion units every year. Despite the efforts to properly dispose of used tires, they still represent a major problem for public health, notably serving as potential breeding sites for vector mosquitoes. Miami-Dade County, Florida has been suffering from arbovirus outbreaks for decades, including dengue, West Nile and yellow fever viruses. The objective of this study was to survey tire shops inserted in the urban matrix across Miami-Dade County for the presence of vector mosquitoes. This study used a cross-sectional design to survey the production of vector mosquitoes at 12 tires shops. Mosquitoes were found in all but one of the tires shops surveyed. We collected a total of 1,110 mosquitoes comprising 528 adults and 582 immatures. *Aedes aegypti* and *Culex quinquefasciatus* were abundantly found in both their immature and adult forms, constituting 99.99% of the mosquito samples collected. *Aedes aegypti* was the most abundant species recorded displaying the highest values in the Shannon and Simpson indices. The findings of this study demonstrate that vector mosquitoes, primarily *Ae*. *aegypti*, are being produced in tires shops in Miami indicating these habitats are highly favorable breeding environments for the production of vector mosquitoes and emphasizing the need to address how the abundance and presence of mosquitoes may vary seasonally in these environments.

## Introduction

Anthropogenic alterations in the environment often promote a non-random biotic homogenization of species, favoring in the process species which are capable of enduring urban environmental conditions and may associate with human activities [1–3].

Important vector species have adapted to and thrive in urban environments, namely *Aedes aegypti* and *Aedes albopictus*, vectors of dengue fever (DENV), chikungunya (CHIKV), yellow fever (YFV) and Zika (ZIKV) viruses [4–8], and *Culex quinquefasciatus* vector of lymphatic filariasis (LF) and West Nile (WNV) and Eastern Equine Encephalitis (EEE) viruses [9, 10].

The increase in urbanization, with more people living in cities than ever before [11], in conjunction with the increasing presence of vector mosquitoes in urban and peri-urban areas put at risk of arbovirus transmission more than half of the world’s population [7, 12–14]. Human mobility in urban environments is a central part of urbanization and has determined the layout of how cites are projected, built and renovated. Most cities in the world were built to accommodate cars, motorcycles and busses [15, 16].

Tires are especially conducive for the production of vector mosquitoes, immature mosquitoes are hidden from predators and the rubber from which tires are made of provides efficient thermal insulation from the elements. Adult mosquitoes also benefit from the tires as they provide adequate and protected resting areas [17]. Tires were also implied responsible for the passive dispersion and further spread of several species of vector mosquitoes [18–20]. It is well documented that *Ae*. *albopictus*, a species of mosquito native from Asia that has spread throughout the globe, mostly due to the commerce of used tires [21–23]. Furthermore, treating tires with insecticide is a complex task, since considerable effort is needed to reach all tires for effective control of a given mosquito population [24, 25].

A major issue associated with urbanization and the vast number of cars is how to properly dispose of used tires, considering the worldwide annual production of approximately 1.4 billion units every year [26]. There have been efforts to recycle used tires, either by the production of fuel [27, 28] or in civil construction [29, 30]. Although there have been efforts to recycle used tires for fuel production or use as construction materials they still represent a major problem for public health.

Controlling vector mosquitoes in urban environments is a great challenge. Insecticide resistance has been reported in important vector species in urban settings [31–33] while the use of alternative novel control methods such as genetically modified mosquitoes is still far from being implemented under the Integrated Vector Management (IVM) framework [34]. Developing reliable mosquito surveillance and control programs capable of detecting insecticide resistance patterns, identify and remove potential breeding sites, and predict fluctuations in the abundance of vector mosquitoes is highly important for controlling the spread of mosquito-borne diseases [35, 36].

The understanding of how mosquitoes explore the physical features present in the urban built environment is crucial to the development of effective long-term control strategies. Therefore, the objective of this study was to survey tires shops across different urban built environments and neighborhoods of Miami-Dade County for the presence of vector mosquitoes.

## Methods

### Study design

**Fig 1:**
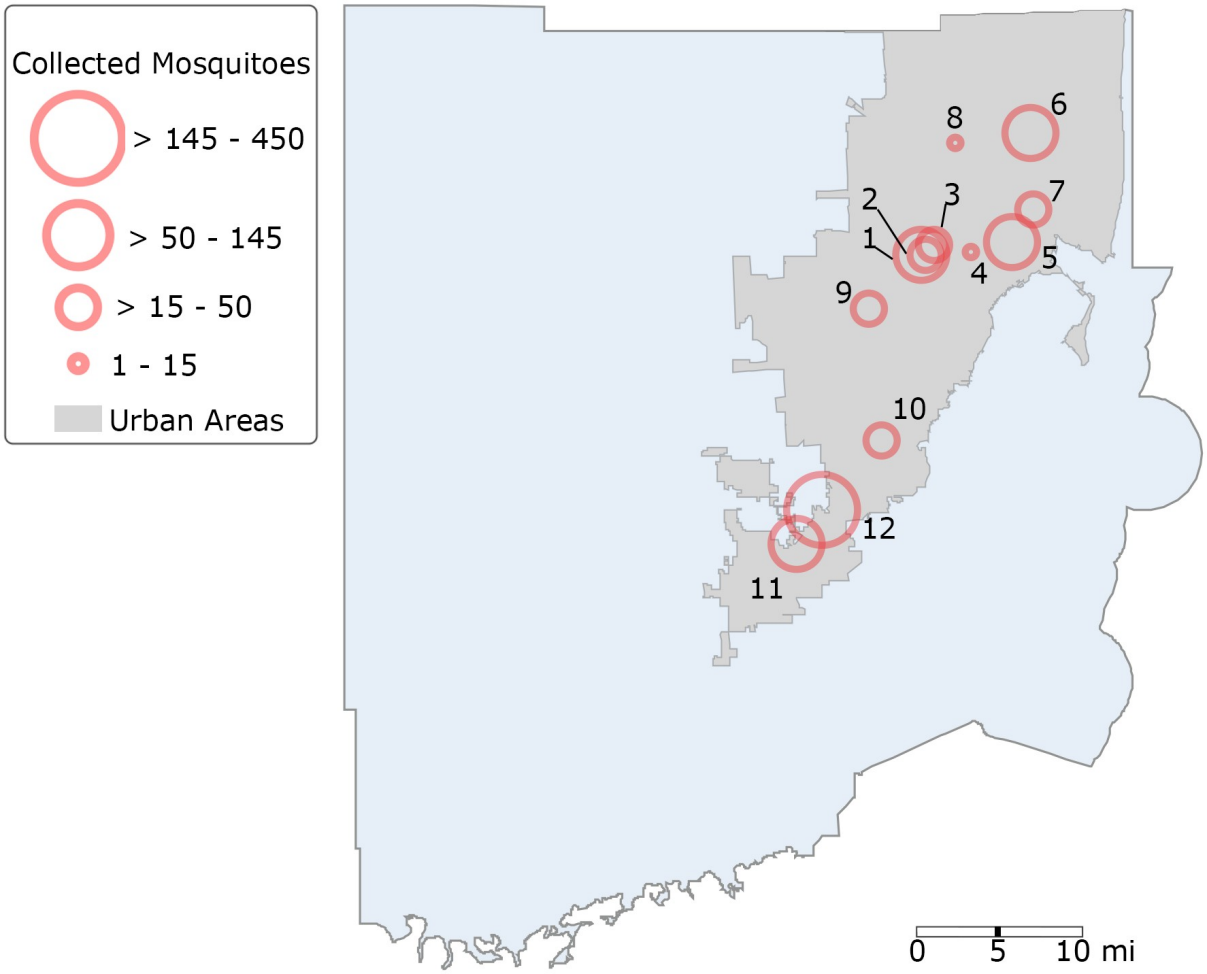
Map showing the location of the mosquito-surveyed tire shops in Miami-Dade, Florida (latitude, 25.761681; longitude, -80.191788). The size of the circles represents the number of mosquitoes collected in each one of the twelve tire shops surveyed. Fig 1 was produced using ArcGIS 10.2 (Esri, Redlands, CA) using freely available layers from the Miami-Dade County’s Open Data Hub—https://gis-mdc.opendata.arcgis.com/.

This study used a cross-sectional design to survey the production of vector mosquitoes in 12 tire shops located in urbanized areas of Miami-Dade County, Florida. Aiming to provide comprehensive coverage of Miami-Dade County, we selected the tires shops among ten different ZIP code regions located throughout Miami-Dade County (Fig 1). Areas previously affected by the Zika virus outbreak in 2016 were also included in this study [37].

### Collection of mosquitoes

Mosquitoes were collected in 2018 during the warmer months of the year in Miami (between July and August), with standardized sampling efforts for all collections. All the tire shops surveyed on this study were located in urban areas and had a similar layout: the main office designed to fit from two to six people, a garage to work and repair from two to six cars, and an outdoor stock of tires ranging from approximately 30 to 200 car tires. The majority of tires were stored outside due to the lack of physical space inside the shops and even when covered were exposed to the elements and collected rainwater. Tires were organized either on stacks of four to ten tires or put side by side on shelves on an upright position (S1 Fig).

Adult mosquitoes were collected with BG-Sentinel traps (Biogents AG, Regensburg, Germany) baited with dry ice [38], for 24 hours. One BG-Sentinel trap was used to collect adult mosquitoes at each tire shop, and when possible, was deployed in a shaded area protected from the elements to enhance the collection of mosquitoes.

Tires were surveyed for immature mosquitoes for two hours or until all potential breeding sites were exhausted. Larvae and pupae were collected with manual plastic pumps (turkey basters) and stored in plastic containers (100 ml) for transport. The collected mosquitoes were transported to the Miami-Dade County Mosquito Control Laboratory and subsequently morphologically identified to species using taxonomic keys [39]. The number of tires in each tire shop was counted for further comparison between the availability of breeding sites and relative abundance of mosquitoes (Table 1).

**Table 1:** Information about the twelve surveyed tire shops in Miami-Dade County, Florida.

Collection Site	Estimated number of tires	Zip code	Collection Year
1	102	33144	2018
2	57	33144	2018
3	51	33144	2018
4	109	33134	2018
5	100	33135	2018
6	83	33150	2018
7	121	33127	2018
8	36	33012	2018
9	30	33010	2018
10	102	33165	2018
11	205	33157	2018
12	202	33032	2018

Since this study posed less than minimal risk to participants and did not involve endangered or protected species the Institutional Review Board at the University of Miami determined that the study was exempt from institutional review board assessment (IRB Protocol Number: 20161212). Collections of mosquitoes in the tire shops were conducted only upon authorization of the owner.

### Data analysis

Biodiversity indices were calculated for all collected mosquitoes based on the Shannon and Simpson (1-D) indices [40, 41], commonly used to evaluate diversity patterns in a community. The Shannon index is used to quantify the number of specimens as well as the number of species, communities with fewer species yield lower values while communities with many species yield higher values. The Simpson (1-D) index is used to assess evenness in a community, in which values closer to 0 denotes that species are equally present while values closer to 1 denote the presence of dominant species [42].

Individual rarefaction curves were generated to estimate both sampling sufficiency and the expected occurrence of species for smaller samples. Plots of cumulative species abundance (ln S), Shannon index (H) and log evenness (ln E) (SHE) profiles were also calculated for the collected mosquitoes; changes in the direction of the lines indicate ecological heterogeneity of mosquito assembly [43]. A bivariate linear regression using ordinary least squares was used to estimate the association between the number of tires and the relative abundance of mosquitoes. Analyses were carried out with 10,000 randomizations without replacement and a 95% confidence interval using Past software (v.3.16) [44, 45].

## Results

Mosquitoes were found in all but one of the 12 tires shops surveyed in this study. We collected a total of 1,110 mosquitoes comprising of 528 adults and 582 immatures, distributed among *Ae*. *albopictus*, *Ae*. *aegypti* and *Cx*. *quinquefasciatus*. Only five adult *Aedes albopictus* were collected in tire shops during this study and no immature was found breeding on tires. On the other hand, *Ae*. *aegypti* and *Cx*. *quinquefasciatus* were abundantly found in both their immature and adult form, comprising 99.99% of the mosquito assembly collected at tires shops.

*Aedes aegypti* was the most abundant mosquito totaling 944 mosquitoes collected, 481 immatures and 463 adults, followed by *Cx*. *quinquefasciatus* with 161 mosquitoes collected, 101 immatures and 60 adults. *Aedes aegypti* was also the most abundant species collected at the pupal stage numbering a total of 58 pupae, being present at all tires shops but one in which no mosquitoes were found (tire shop 4). *Culex quinquefasciatus* was only found the pupal stage at two tire shops (Table 2, S1 Fig).

**Table 2:** Total number of mosquitoes collected at tire shops in Miami-Dade County, Florida.

Tire Shop	*Aedes aegypti*		*Culex quinquefasciatus*		*Aedes albopictus*	
	Immature	Adult	Immature	Adult	Immature	Adult
1	14 (2)		73 (5)			
2	25 (6)	9		3		
3	19 (6)	8		9		
4	0	0		0		
5	67 (4)	11	5	0		
6	77 (11)	55		2		
7	33 (6)	9		4		
8	12 (3)	0		0		
9	31 (8)	6	3	1		1
10	10 (3)	22		2		
11	70 (4)	3	11 (2)	1		4
12	65 (5)	340	2	38		
Total	423 (58)	463	94 (7)	60		5

In parentheses, the number of pupae collected.

The bivariate linear regression used to estimate the association between the number of tires and the relative abundance of mosquitoes was significant when all collected mosquitoes were included in the analysis (r = 0.64; r2 = 0.41; *P* = 0.02). However, when species were analyzed individually only *Ae*. *aegypti* yielded significant results (r = 0.63; r2 = 0.39; *P* = 0.03). The lack of significant values for both *Cx*. *quinquefasciatus* and *Ae*. *albopictus* indicate that there is no association between the availability of tires and the relative abundance of these species (Table 3).

**Table 3:** Bivariate linear regression for mosquitoes collected at tires shops in Miami-Dade County, Florida.

Species	r	r^2^	*P-*value
*Aedes aegypti*	0.6313	0.39854	**0.037238**
*Culex quinquefasciatus*	0.31586	0.099767	0.37396
*Aedes albopictus*	0.47661	0.22716	0.11722
All collected mosquitoes	0.64176	0.41186	**0.024469**

In bold are significant values.

The average value for the Shannon’s diversity index for all collected mosquitoes was 1.299 (95% CI: 1.217–1.425). *Aedes aegypti* yielded the highest value 1.854 (95% CI: 1.783–1.915), followed by *Cx*. *quinquefasciatus*, 1.545 (95% CI: 1.370–1.688) and *Ae*. *albopictus* 0.500 (95% CI: 0.500–0.673). Similar results were also found for the Simpson (1-D) index, *Ae*. *aegypti* yielded the highest value 0.760 (95% CI: 0.735–0.781), followed by *Cx*. *quinquefasciatus*, 0.690 (95% CI: 0.627–0.740) and *Ae*. *albopictus* 0.320 (95% CI: 0.320–0.480) (S2 Fig).

**Fig 2:**
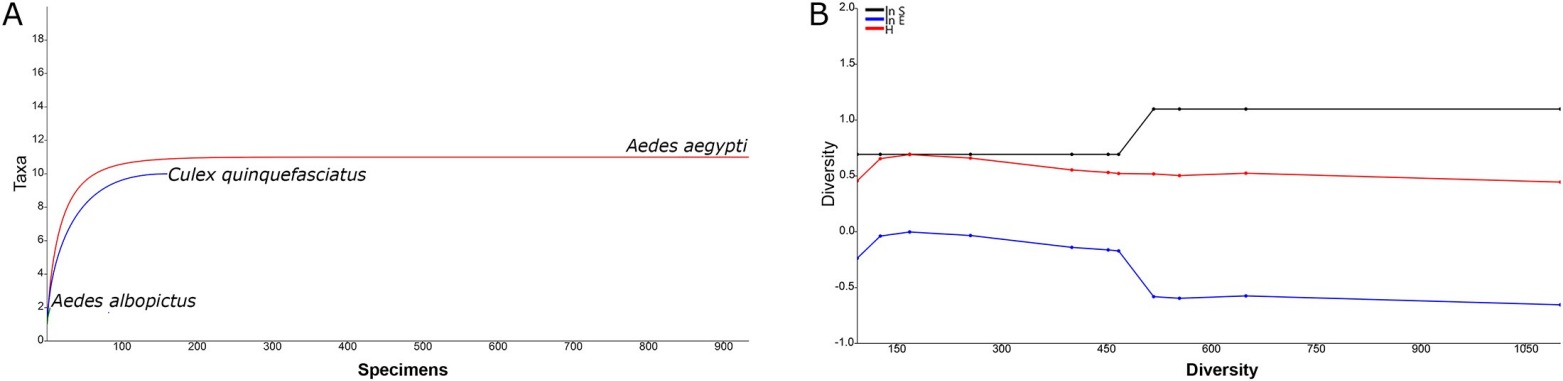
Biodiversity indices for mosquitoes collected at tires shops in Miami-Dade County, Florida. (A) Individual rarefaction curves; (B) Plots of cumulative SHE profiles (ln S, H and ln E).

The individual rarefaction curves indicated that sampling sufficiency was highly asymptotic for *Ae*. *aegypti* with a considerable degree of confidence for predicting the presence of this species for smaller sample sizes. The rarefaction curve of *Cx*. *quinquefasciatus*, on the other hand, did not reach asymptotic stability, nor did *Ae*. *albopictus*. The lack of substantial changes in the cumulative SHE analysis indicates the lack of heterogeneity regarding species composition, diversity and evenness for the mosquitoes found breeding at tires shops (Fig 2).

## Discussion

This study indicated that *Ae*. *aegypti* is the most abundant species breeding at tires shops in Miami-Dade County. *Aedes aegypti* immature and adult mosquitoes were collected in all tire shops apart from one. Although adult mosquitoes collected at the tires shops could have emerged from a number of other sources within the community the detection of pupae clearly indicates efficient production of *Ae*. *aegypti* mosquitoes in these environments as the presence of pupae in a given breeding site can be used as a proxy for estimating adult mosquito production [46].

*Culex quinquefasciatus* was the second most abundant species being found both in larval and pupal and adult stages. Only five adult *Ae*. *albopictus* were collected during this study and were considered as accidental catches. Results from the bivariate linear regression were only significant for *Ae*. *aegypti* indicating that there is a positive association between the number of tires and its relative abundance.

Even small tires shops with no more than 30 tires in stock were producing *Ae*. *aegypti* in large numbers. The widespread presence of *Ae*. *aegypti* in tire shops located in highly urbanized areas with restrict availability of sugar sources and reduced biodiversity of potential hosts increase their contact with humans [47]. Vector mosquitoes such as *Ae*. *aegypti* are known for not having a great dispersal ability [48], and even when collections were done at tire shops close to each other, the results differed significantly. The detection of *Ae*. *aegypti* pupae in all tire shops apart from one clearly indicates efficient production of *Ae*. *aegypti* mosquitoes in these environments, highlighting how this species benefits from urbanization and subsequent species homogenization [25, 49–51].

Miami is a multi-cultural city with a high flow of people traveling from and to endemic areas, especially from the Caribbean and Latin America [52]. Miami has been tormented by arbovirus outbreaks for decades, including DENV, WNV and YFV [53–58]. More recently, during the 2016 ZIKV outbreak it was the most affected area in the continental United States [37, 59], exposing the unquestionable vulnerability of Miami to the introduction of arboviruses.

Human mobility is an important risk factor for increasing prevalence and incidence in recent decades by significantly increasing the prevalence and incidence of vector-borne diseases since travelers from endemic areas may involuntarily bring pathogens to their destinations infecting local mosquito vectors [60]. During the ZIKV outbreak in Miami in 2016, several introductions of the virus were documented [59], exposing at the same time the vulnerability of Miami to the introduction, establishment and local transmission of arboviruses and the importance of controlling populations of *Ae*. *aegypti* in populated urban areas [61].

Tire shop workers spend a disproportional amount of time outdoors and are particularly vulnerable to bites of vector mosquitoes and, therefore, are subjected to an elevated risk of being exposed to arboviruses. Moreover, tires shops are often small family companies and do not follow strict safety or best practice guidelines, which may result in higher production of mosquitoes and, as a consequence, increased exposure to potential infective bites and arboviruses.

The trade and commerce of used tires, especially those from the highly productive environment such as the tires shops may inadvertently disperse mosquitoes to other areas [19, 20, 23]. Eggs of *Ae*. *aegypti* are especially resistant and can survive in a latent state for months while waiting for the right conditions to hatch [62]. As tire shops in Miami-Dade County are productive breeding foci, especially for *Ae*. *aegypti*, the potential exportation of *Ae*, *aegypti* eggs to other areas of the city or even different cities and states should be investigated.

The only tires shop surveyed on this study that was not producing vector mosquitoes (Tire Shop 4) was very well kept with all tires adequately covered and dry. The remaining of the tires shops surveyed in this study were producing mosquitoes mostly due to the lack of awareness or negligence, despite considerable efforts made by the Miami-Dade Mosquito Control Division in educating the population on the importance of eliminating potential breeding sites. Such a scenario highlights the need for improved guidelines and further enforcing to reduce the role of tire shops in the production of vector mosquitoes in Miami-Dade.

Data collection across all weather and season variations will further enhance our insight regarding tire shops and vector abundance.

## Conclusions

The main findings of this study demonstrate that vector mosquitoes, primarily *Ae*. *aegypti*, are being produced at tire shops in Miami-Dade County. Such findings suggest that tire shops have a significant role in the production of vector mosquitoes in Miami and that more studies are needed to address how the abundance and presence of mosquitoes may vary seasonally in these environments in light of mosquito and disease control.

## Supporting information

S1 Fig Example of the conditions found in tires shops in Miami-Dade County, Florida. (A) Tires stored outside exposed to the elements; (B) *Aedes aegypti* larvae breeding inside a tire; and (C) *Aedes aegypti* pupae inside a tire, indicated by black arrow.
(TIF)

S2 Fig Shannon (A) and Simpson (B) indices for mosquitoes collected at tires shops in Miami-Dade County, Florida. (TIF)

## Abbreviations

CHIKV: Chikungunya Virus
DENV: Dengue Fever Virus
EEE: Eastern Equine Encephalitis
IVM: Integrated Vector Management
LF: Lymphatic Filariasis
YFV: Yellow Fever Virus
WNV: West Nile Virus
ZIKV: Zika Virus
