# Supplementary figures and images for: Tire shops in Miami-Dade County, Florida are important producers of vector mosquitoes
Source: PLoS One. 2019 May 20;14(5):e0217177. doi: 10.1371/journal.pone.0217177 (PMC6527201; doi:10.1371/journal.pone.0217177)

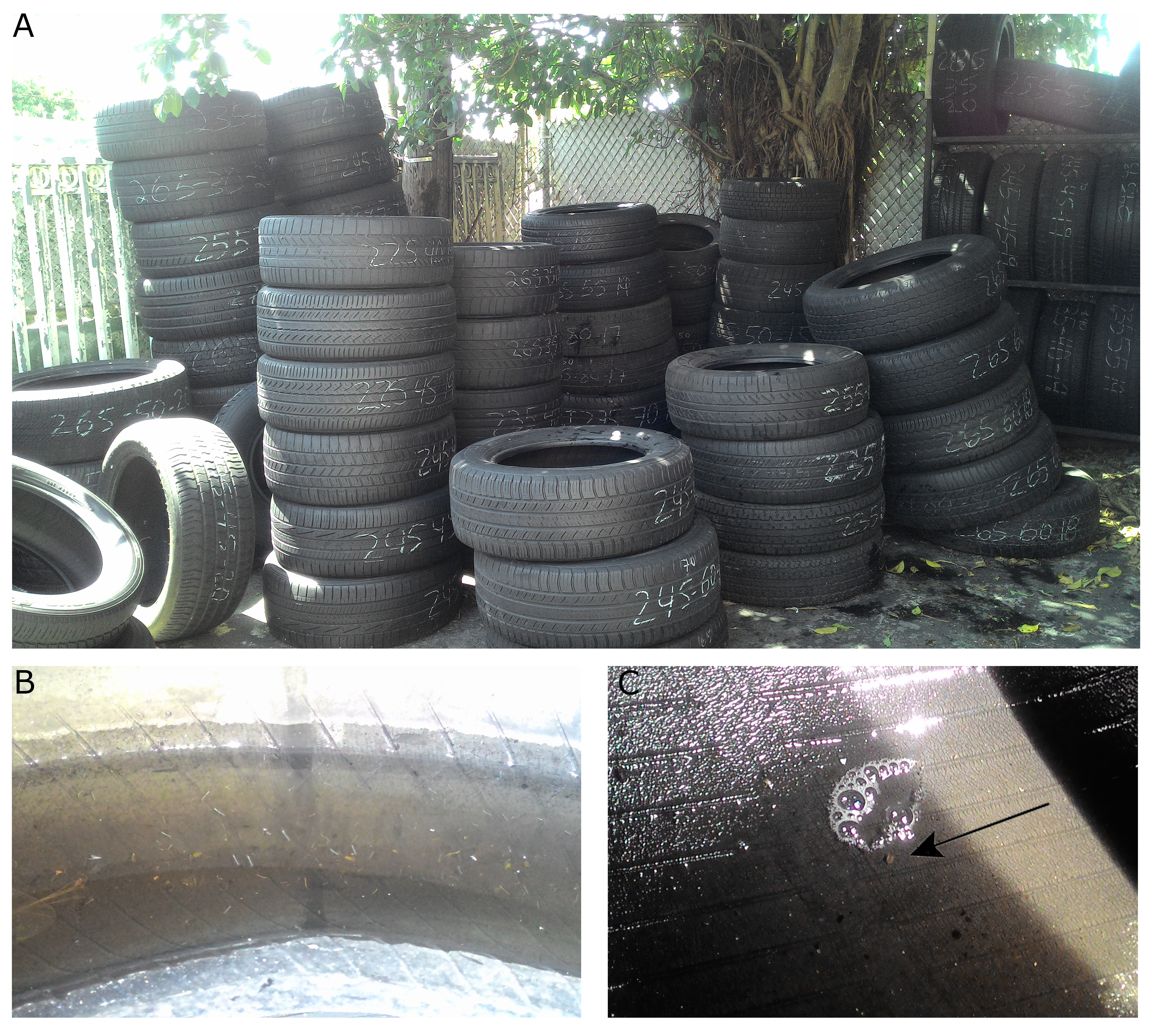

Supplement: S1 Fig — (A) Tires stored outside exposed to the elements; (B) Aedes aegypti larvae breeding inside a tire; and (C) Aedes aegypti pupae inside a tire, indicated by black arrow. (TIF) [file pone.0217177.s001.tif]

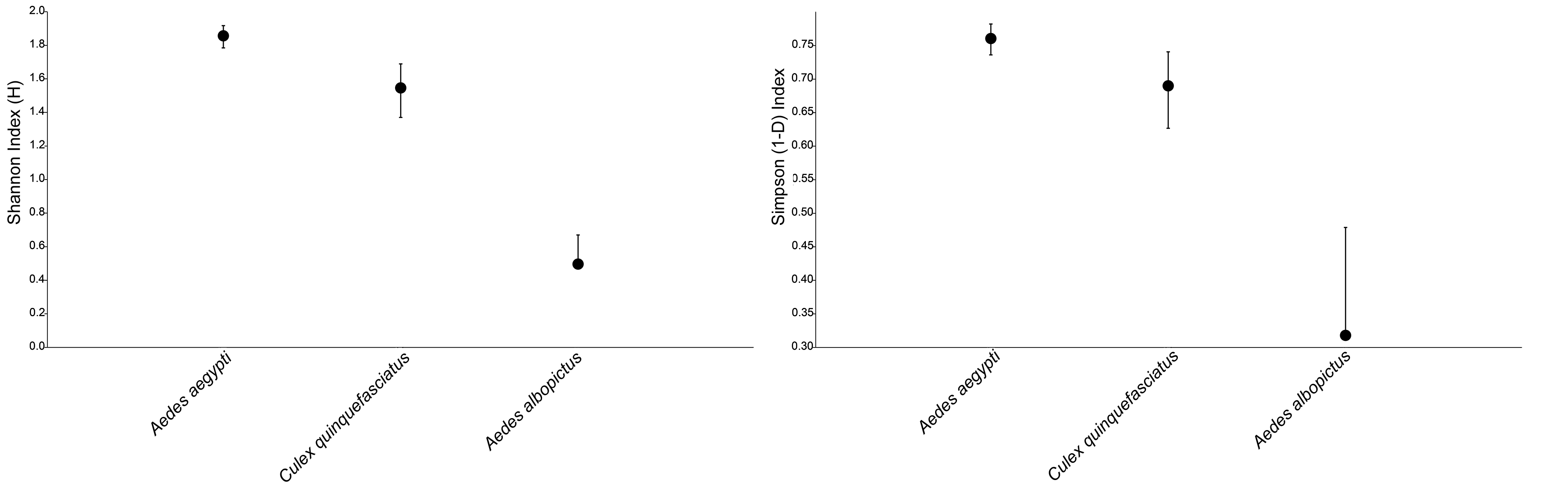

Supplement: S2 Fig — (TIF) [file pone.0217177.s002.tif]
